# Supplementary material for: Technical and Methodological Aspects of Cell-Free Nucleic Acids Analyzes
Source: Int J Mol Sci. 2020 Nov 16;21(22):8634. doi: 10.3390/ijms21228634 (PMC7697251; doi:10.3390/ijms21228634)
Supplement: Supplementary file 1 [file ijms-21-08634-s001.zip › ijms-943979-supplementary.docx]

**Table 1.** **Concentrations of cfNAs in different human body fluids.** In several studies quantities of genetic material were expressed as genome equivalents per milliliter, in such cases, we calculated the amount of DNA with a conversion factor of 6.6 pg of DNA equal to 1 genome equivalent. Interquartile range (IQR).

NA - Not available.

*Peritoneal dialysis patients without any history of systemic inflammation or peritonitis in the last 3 months.

**Insufficient methodology data.

| **Phenotype** | **Type of cfNAs** | **Source** | **Samples** | **Concentration** | **Reference** |
| --- | --- | --- | --- | --- | --- |
| Gastric cancer | cfDNA | Plasma | 130 | 68.7 ± 40.5 ng/ml | [[1]](https://paperpile.com/c/33ouGt/z5al) |
| Gastric cancer | cfDNA | Serum | 130 | 755.9 ± 1203.7 ng/ml | [[1]](https://paperpile.com/c/33ouGt/z5al) |
| Healthy | cfDNA | Saliva | 26 | 1.11 (0.01–67.63) ng/ml | [[2]](https://paperpile.com/c/33ouGt/0Apc) |
| Pulmonary benign disease | cfDNA | Saliva | 15 | 3.26 (0.029–26.30) ng/ml | [[2]](https://paperpile.com/c/33ouGt/0Apc) |
| Non-small-cell lung carcinoma | cfDNA | Saliva | 68 | 0.531 (0.018–285.420) ng/ml | [[2]](https://paperpile.com/c/33ouGt/0Apc) |
| Healthy | cfDNA | Urine | 19 | 6–50 ng/ml | [[3]](https://paperpile.com/c/33ouGt/d3Kh) |
| Prostate cancer | cfDNA | Seminal plasma | 6 | 2243670 ± 1758000 ng/ml | [[4]](https://paperpile.com/c/33ouGt/XxSj) |
| Healthy | cfDNA | Seminal plasma | 3 | 57700 ± 4800 ng/ml | [[4]](https://paperpile.com/c/33ouGt/XxSj) |
| Normozoospermia | cfDNA | Seminal plasma | 11 | 1340 ± 650 (510 to 2730) ng/ml | [[5]](https://paperpile.com/c/33ouGt/mJZH) |
| Azoospermia | cfDNA | Seminal plasma | 9 | 2560 ± 1430 (820 to 5450) ng/ml | [[5]](https://paperpile.com/c/33ouGt/mJZH) |
| Healthy | cfDNA | Tear fluid | 11 | 1400 (200) ng/ml | [[6]](https://paperpile.com/c/33ouGt/m9Sw) |
| Non-autoimmune dry eye disease | cfDNA | Tear fluid | 13 | 2900 (600) ng/ml | [[6]](https://paperpile.com/c/33ouGt/m9Sw) |
| Autoimmune dry eye disease | cfDNA | Tear fluid | 11 | 5200 (1200) ng/ml | [[6]](https://paperpile.com/c/33ouGt/m9Sw) |
| Graft versus host disease | cfDNA | Tear fluid | 12 | 9100 (2300) ng/ml | [[6]](https://paperpile.com/c/33ouGt/m9Sw) |
| Healthy | cfDNA | Sweat | 10 | 11.5 ng/ml | [[7]](https://paperpile.com/c/33ouGt/gP1d) |
| Lung cancer | cfDNA | Sputum | 28 | 312 ± 61 ng/ml | [[8]](https://paperpile.com/c/33ouGt/WKxy) |
| Healthy | cfDNA | Sputum | 68 | 1033 ± 227 ng/ml | [[8]](https://paperpile.com/c/33ouGt/WKxy) |
| Cancer | cfDNA | Ascites | 6 | 3.9–38.4 ng/ml | [[9]](https://paperpile.com/c/33ouGt/QhYO) |
| Cirrhosis | cfDNA | Ascites | 1 | 8.35 ng/ml | [[9]](https://paperpile.com/c/33ouGt/QhYO) |
| Rheumatoid arthritis | cfDNA | Synovial fluid | 80 | 3,182 ng/ml | [[10]](https://paperpile.com/c/33ouGt/xV29) |
| Osteoarthritis | cfDNA | Synovial fluid | 33 | 82 ng/ml | [[10]](https://paperpile.com/c/33ouGt/xV29) |
| Rheumatoid arthritis | cfDNA | Plasma | 80 | 41.3 ng/ml | [[10]](https://paperpile.com/c/33ouGt/xV29) |
| Healthy | cfDNA | Plasma | 50 | 32.1 ng/ml | [[10]](https://paperpile.com/c/33ouGt/xV29) |
| Peritoneal dialysis control* | cfDNA | Peritoneal effluent | 30 | 0.2 (IQR 0.1–0.3) ng/ml | [[11]](https://paperpile.com/c/33ouGt/S1b9) |
| Peritoneal dialysis | cfDNA | Peritoneal effluent | 23 | 168.5 (IQR 124.3–3033) ng/ml | [[11]](https://paperpile.com/c/33ouGt/S1b9) |
| Continuous ambulatory peritoneal dialysis | cfDNA | Peritoneal effluent | 18 | 1.8–9.5 ng/ml | [[12]](https://paperpile.com/c/33ouGt/jaBY) |
| Lung adenocarcinoma | cfDNA | Bronchoalveolar lavage fluid | 20 | 5490–20130 ng/ml | [[13]](https://paperpile.com/c/33ouGt/CJOa) |
| High risk pancreatic cyst | cfDNA | Cyst fluid | 10 | 44463±39228 ng/ml | [[14]](https://paperpile.com/c/33ouGt/8VII) |
| Low risk pancreatic cyst | cfDNA | Cyst fluid | 20 | 33021±20004 ng/ml | [[14]](https://paperpile.com/c/33ouGt/8VII) |
| Healthy | cfDNA | Serum | 19 | 20±3 ng/ml | [[14]](https://paperpile.com/c/33ouGt/8VII) |
| Pancreatic cancer | cfDNA | Serum | 19 | 36±14 ng/ml | [[14]](https://paperpile.com/c/33ouGt/8VII) |
| Retinoblastoma | cfDNA | Aqueous humour | 12 | 140–394000 (median 1670) ng/ml | [[15]](https://paperpile.com/c/33ouGt/Ls9U) |
| Retinoblastoma | cfDNA | Aqueous humour | 6 | 84–56000 (median 174) ng/ml | [[16]](https://paperpile.com/c/33ouGt/vukL) |
| Central nervous system cancer | cfDNA | Cerebrospinal fluid | 27 | 8440 (100–110000) ng/ml | [[17]](https://paperpile.com/c/33ouGt/dVV2) |
| Pregnant woman with male fetus | cffDNA | Cerebrospinal fluid | 17 | 17.61 (1.51–83.58) ng/ml | [[18]](https://paperpile.com/c/33ouGt/TYGx) |
| Retinoblastoma | cf-miRNA | Aqueous humour | 6 | 79–150000 (median 273) ng/ml | [[16]](https://paperpile.com/c/33ouGt/vukL) |
| Healthy | cfRNA | Saliva | 5 | 1945 (IQR 2495) ng/ml | [[19]](https://paperpile.com/c/33ouGt/nPnE) |
| Healthy | cfRNA | Plasma | 4 | 44 (41–51) ng/ml | [[20]](https://paperpile.com/c/33ouGt/sHSQ) |
| Small-cell and non-small-cell lung carcinoma | cfRNA | Plasma | 10+10 | 400 (109–1228) ng/ml | [[20]](https://paperpile.com/c/33ouGt/sHSQ) |
| Healthy | cfRNA | Plasma | 5 | 308 (IQR 104) ng/ml | [[19]](https://paperpile.com/c/33ouGt/nPnE) |
| Healthy | cfRNA | Urine | 19 | 24–140 ng/ml | [[3]](https://paperpile.com/c/33ouGt/d3Kh) |
| Healthy | cfRNA | Seminal plasma | 10 | 1750 (870–3640) ng/ml | [[21]](https://paperpile.com/c/33ouGt/i9Mv) |
| Healthy | cfRNA | Seminal plasma | NA** | 17770 (QRI 7673) ng/ml | [[19]](https://paperpile.com/c/33ouGt/nPnE) |
| Healthy | cfRNA | Tear fluid | 5 | 564 (IQR 631) ng/ml | [[19]](https://paperpile.com/c/33ouGt/nPnE) |
| Healthy | cfRNA | Pleural fluid | 5 | 470 (IQR 190) ng/ml | [[19]](https://paperpile.com/c/33ouGt/nPnE) |
| Healthy | cfRNA | Bronchial lavage fluid | 5 | 1128 (IQR 886) ng/ml | [[19]](https://paperpile.com/c/33ouGt/nPnE) |
| Healthy | cfRNA | Breast milk | NA** | 47240 (IQR 73180) ng/ml | [[19]](https://paperpile.com/c/33ouGt/nPnE) |
| Healthy | cfRNA | Colostrum | 1 | 585 ng/ml | [[19]](https://paperpile.com/c/33ouGt/nPnE) |
| Healthy | cfRNA | Urine | 5 | 94 (IQR 129) ng/ml | [[19]](https://paperpile.com/c/33ouGt/nPnE) |
| Healthy | cfRNA | Peritoneal effluent | 5 | 775 (IQR 345) ng/ml | [[19]](https://paperpile.com/c/33ouGt/nPnE) |
| Central nervous system cancer | cfRNA | Cerebrospinal fluid | 24 | 4210 (300–40600) ng/ml | [[17]](https://paperpile.com/c/33ouGt/dVV2) |
| Healthy | cfRNA | Cerebrospinal fluid | 5 | 111 (IQR 66) ng/ml | [[19]](https://paperpile.com/c/33ouGt/nPnE) |
| Healthy | cfRNA | Amniotic fluid | NA** | 570 (IQR 354) ng/ml | [[19]](https://paperpile.com/c/33ouGt/nPnE) |
| Retinoblastoma | cfRNA | Aqueous humour | 6 | 400–12800 (median 653) ng/ml | [[16]](https://paperpile.com/c/33ouGt/vukL) |
| HIV‐positive | HIV‐RNA | Cervico‐vaginal secretion | 40 | 250 copies/ml | [[22]](https://paperpile.com/c/33ouGt/Ak7S) |
| HIV-seropositive pregnant | HIV‐RNA | Cervico‐vaginal secretion | 26 | ~550 copies/ml | [[23]](https://paperpile.com/c/33ouGt/RJ4f) |

**Table 2.** **The size range of cfNAs in different human body fluids.** Based on the studies, we summarise the length of the most abundant fraction of cfNAs fragments, however both shorter and longer fragments could also be present in body fluids (not included).

| **Phenotype** | **Type of cfNAs** | **Source** | **Samples** | **Size** | **Reference** |
| --- | --- | --- | --- | --- | --- |
| Cancer | cfDNA | Plasma | 51 | 155 bp | [[24]](https://paperpile.com/c/33ouGt/Jeq7) |
| Cancer | cfDNA | Serum | 46 | 150–2000 bp | [[24]](https://paperpile.com/c/33ouGt/Jeq7) |
| Healthy | cfDNA | Urine | 19 | main part 150–400 bp (up to 19000 bp in female) | [[3]](https://paperpile.com/c/33ouGt/d3Kh) |
| Normozoospermia, Azoospermia | cfDNA | Seminal plasma | 11+9 | 180 bp–15000 bp | [[5]](https://paperpile.com/c/33ouGt/mJZH) |
| Non-small-cell lung carcinoma | cfDNA | Plasma | 50 | ~160 bp | [[25]](https://paperpile.com/c/33ouGt/F1ml) |
| Non-small-cell lung carcinoma | cfDNA | Sputum | 50 | ~160 bp | [[25]](https://paperpile.com/c/33ouGt/F1ml) |
| Non-small-cell lung carcinoma | cfDNA | Urine | 50 | ~100 bp | [[25]](https://paperpile.com/c/33ouGt/F1ml) |
| Healthy | cfDNA | Urine | 5 | 150–250 bp | [[26]](https://paperpile.com/c/33ouGt/yOOl) |
| Biliary tract carcinoma | cfDNA | Bile | 10 | ~6000 bp | [[27]](https://paperpile.com/c/33ouGt/MoiH) |
| Cancer | cfDNA | Ascites | 6 | main peak 150–160 bp; smaller peak 300–400 bp | [[9]](https://paperpile.com/c/33ouGt/QhYO) |
| Lung adenocarcinoma | cfDNA | Bronchial lavage fluid | 20 | ~10000 bp | [[13]](https://paperpile.com/c/33ouGt/CJOa) |
| Enucleated eyes | cfDNA | Aqueous humour | 12 | ~133 bp | [[15]](https://paperpile.com/c/33ouGt/Ls9U) |
| Enucleated eyes | cfDNA | Aqueous humour | 6 | 145–165 (median 150 bp) | [[16]](https://paperpile.com/c/33ouGt/vukL) |
| Healthy | cfDNA | Breast milk | NA | ~180 bp | [[28]](https://paperpile.com/c/33ouGt/K4Oa) |
| Pregnant woman | cffDNA | Urine | 5 | 29–45 bp | [[29]](https://paperpile.com/c/33ouGt/Q0IH) |
| Pregnant woman | cfDNA | Urine | 7 | <100 bp | [[29]](https://paperpile.com/c/33ouGt/Q0IH) |

**Table 3.** **The list of collection tubes or stabilization solutions developed for the preservation of cfNAs from different body fluids.** In the table there are summarized different preservation solutions (collection tubes, collection devices or stabilization buffers) for the most known body fluids. Each of the solutions contain information regarding its suitability for the type of the genetic material together with the manufacturer's recommendations for the storage time and temperature and also information about compatibility with downstream analysis. There is also a mention about the preservation reagents, however, in many cases it is proprietary information.

RT - room temperature.

NA - not available.

* Web page does not work anymore.

| **Specimen** | **Manufacturer** | **Preservation solution** | **Type of cfNAs** | **Storage time** | **Temperature** | **Reagents** | **Sufficient for downstream analysis and applications** | **References** |
| --- | --- | --- | --- | --- | --- | --- | --- | --- |
| Blood | Streck (La Vista, NE 68128 USA) | Cell-Free DNA BCT® | cfDNA | 14 days | 6-37 °C | anticoagulant K_3_EDTA and a cell preservative in a liquid medium | a wide range of downstream applications | [[30]](https://paperpile.com/c/33ouGt/23Vt) |
|  |  |  | CTCs | 7 days | 15-30 °C |  |  |  |
|  |  | RNA Complete BCT™ | cfRNA | 7 days | RT | anticoagulant K_3_EDTA and a cell preservative in a liquid medium | qualitative and quantitative real-time PCR, droplet digital PCR, NGS, and Nanoparticle Tracking Analysis. |  |
|  |  |  | extracellular vesicles (exosomes) | 7 days | RT |  |  |  |
|  | CellSearch (Menarini Silicon Biosystems Inc 3401 Masons Mill Road, Suite 100 Huntington Valley, PA) | CellSave Preservative Tubes | CTCs | 4 days | RT | proprietary preservative agent | NA | [[31]](https://paperpile.com/c/33ouGt/aRRr) |
|  | Qiagen  (PreAnalytiX GmbH  Feldbachstrasse  8634 Hombrechtikon  Switzerland) | PAXgene Blood ccfDNA Tubes | cfDNA | 10 days | RT | proprietary stabilization reagent | PCR, including digital, multiplex and quantitative real-time PCR, methylation-based assays, pharmacogenomic studies, SNP genotyping, NGS | [[32]](https://paperpile.com/c/33ouGt/Xtg6) |
|  |  |  |  | 7 days | 30 °C |  |  | [[33]](https://paperpile.com/c/33ouGt/L2i8) |
|  |  |  |  | 3 days | 37 °C |  |  |  |
|  | Roche (F. Hoffmann-La Roche Ltd. CH-4070 Basel, Switzerland) | Cell-Free DNA Collection Tube® | cfDNA | 7 days | 4 °C, RT, 37 °C | anticoagulant K_3_EDTA and a cell preservative | NA | [[34]](https://paperpile.com/c/33ouGt/TxyL) |
|  | Norgen (3430 Schmon Parkway, Thorold, ON, Canada, L2V 4Y6) | cf-DNA/cf-RNA Preservative Tubes | cfDNA | 30 days | RT | proprietary anticoagulant and non formaldehyde proprietary preservation agent | PCR, qPCR, rt-qPCR, methylation-sensitive PCR, Southern blot analysis, gene expression analysis, microarrays and NGS. | [[35]](https://paperpile.com/c/33ouGt/O3os) |
|  |  |  |  | 8 days | 37 °C |  |  |  |
|  |  |  | ctDNA | 30 days | RT |  |  |  |
|  |  |  |  | 8 days | 37 °C |  |  |  |
|  |  |  | cfRNA | 30 days | RT |  |  |  |
|  |  |  | CTCs | 14 days | RT |  |  |  |
|  | Biomatrica (5627 Oberlin Drive, Suite 120 San Diego, CA 92121) | LBgard® Blood Tubes | cfDNA | 7 days | 4-25 °C | proprietary reagent | digital PCR, NGS, methylation assays, imaging, immunotyping, prenatal testing, pharmacogenomics, genotyping, cancer diagnostics, allograft acceptance | [[36]](https://paperpile.com/c/33ouGt/7uo5) |
|  |  |  | CTCs | 4 days | 18-30 °C |  |  |  |
|  | ProTeck (CFGenome LLC, Denver, CO, USA) | Blood Exo DNA ProTeck® | cfDNA | NA | NA | stabilizing reagent very similar to BCTs by Streck; CFGenome is not providing this product anymore | NA | [[37]](https://paperpile.com/c/33ouGt/51WQ) |
|  |  |  | cffDNA |  |  |  |  |  |
|  | MagBio Genomics Inc. (944 Clopper Road  Gaithersburg, MD 20878 USA) | Blood STASIS™ 21-cfDNA Blood Collection Tubes | cfDNA | 21 days | 15-30 °C | proprietary stabilizing reagent | NA | [[38]](https://paperpile.com/c/33ouGt/NEIz) |
|  |  |  | CTCs | 7 days | 4-37 °C | proprietary non-fixative additive |  |  |
|  | Biocept (5810 Nancy Ridge Dr., Suite 150 San Diego, CA 92121) | CEE-Sure™ BCT | cfDNA | 8 days | 6-37 °C | anticoagulant ACD-A (Acid Citrate Dextrose) and a Formaldehyde Releasing Reagent | NA | [[39]](https://paperpile.com/c/33ouGt/BD7f) |
|  |  |  | CTCs | 4 days | 18-25 °C |  |  |  |
|  | Inresearch Medical Limited (TCL Tower, No.8 Tai Chung Rd, Tsuen Wan, Hongkong) | ImproGene™ Cell Free DNA Tube | cfDNA | 7-14 days | 4-30 °C | anticoagulant with proprietary preservative | NA | [[40]](https://paperpile.com/c/33ouGt/wsML)* |
|  | EONE-DIAGNOMICS Genome Center  (#291 Harmony-ro, Yeonsu-gu, Incheon, 22014, South Korea) | NICE® Check cfDNA Tube | cfDNA | NA | NA | NA | NA | [[41]](https://paperpile.com/c/33ouGt/ZWlb) |
| Saliva | Norgen (3430 Schmon Parkway, Thorold, ON, Canada, L2V 4Y6) | Saliva Exosome Collection and Preservation Kit | exosomes, cfRNA | 2 years | RT | proprietary preservative | NA | [[42]](https://paperpile.com/c/33ouGt/aIUn) |
|  | Oasis Diagnostics® Corporation (15720 NE 31st Avenue,  Vancouver, WA 98686 USA) | Pure•SAL™  Ideal for Liquid Biopsy and Exosomes | cfDNA, cfRNA, exosomes, proteins | NA | NA | proprietary patented  device | PCR, genotyping, sequencing, proteomics and other applications, depending upon the desired results | [[43]](https://paperpile.com/c/33ouGt/gDXV) |
|  |  | RNAPro•SAL™  Split Sample Kit for Liquid Biopsy | RNA, cfDNA, cfRNA, exosomes | NA | NA | proprietary patented  device | not specified |  |
| Urine | Streck (La Vista, NE 68128 USA) | Cell-Free DNA Urine Preserve | cfDNA | 7 days | 6-37 °C | urine enzyme inhibitors and a cell  preservative and PCR compatible blue dye | qPCR, ddPCR, and other methods used to profile circulating DNA. | [[44]](https://paperpile.com/c/33ouGt/gsxi) |
|  | Hunan UPSBio Inc., (Hunan University National Science Park, Changsha, Hunan, China) | Urine Collection Tube | cfDNA | 7 days | RT | proprietary chemicals from Hunan UPSBio, Inc. | NA | NA |
|  | Zymo Research (IRVINE 17062 Murphy Ave. Irvine, CA 92614, U.S.A.) | Urine Conditioning Buffer (UCB) | DNA, RNA, cfDNA | 1 month | RT | NA | NA | [[45]](https://paperpile.com/c/33ouGt/AoRo) |
|  | Norgen (3430 Schmon Parkway, Thorold, ON, Canada, L2V 4Y6) | Urine Preservation | DNA, RNA, miRNA, proteins, cf-miRNA | 2 years | RT | NA | NA | [[46]](https://paperpile.com/c/33ouGt/wAv7) |
|  | DNA Genotek Inc. (3000 - 500 Palladium Drive Ottawa, Ontario, Canada  K2V 1C2) | Colli-Pee® | not specified | 7 days | RT | the non-toxic Novosanis proprietary Urine Conservation Medium (UCM) | NA | [[47]](https://paperpile.com/c/33ouGt/dMKw) |
| Stool | Zymo Research  (IRVINE 17062 Murphy Ave.  Irvine, CA 92614, U.S.A.) | DNA/RNA Shield Fecal Collection tubes | DNA | 2 years | 4-25 °C | proprietary preservative | NA | [[48]](https://paperpile.com/c/33ouGt/E4JG) |
|  |  |  | RNA | 1 month | 4-25 °C |  |  |  |
|  | Norgen (3430 Schmon Parkway, Thorold, ON, Canada, L2V 4Y6) | Stool Nucleic Acid Collection and Preservation Tubes | DNA | 2 years | RT | Norgen’s Stool Preservative in a liquid format | NA | [[49]](https://paperpile.com/c/33ouGt/b8iO) |
|  |  |  | RNA | 7 days | RT |  |  |  |
|  | DNA Genotek (Kanata, ON, Canada) | OMNIgene•GUT | DNA | 1 month | RT | ~2 ml of a proprietary buffer and a large stainless steel bead | 16S rRNA microbiome profiling, shotgun metagenomic sequencing, qPCR and arrays | [[50]](https://paperpile.com/c/33ouGt/5Vga) |

**Table 4. The list of methods developed for the isolation of exosomes from different body fluids.** In the table there are summarized different procedures developed for the extraction of exosomes from various body fluids. They are sorted based on the type of the body fluid from which they were extracted.

| **Source** | **Kit name (Manufacturer)** | **Type** | **References** |
| --- | --- | --- | --- |
| **Serum** | exo-Spin (Cell Guidance Systems) | Size exclusion-based | [[51]](https://paperpile.com/c/33ouGt/h0Dx) |
|  | exoEasy Maxi Kit (Qiagen) | Spin column | [[52]](https://paperpile.com/c/33ouGt/Mb2P) |
|  | Capturem Extracellular Vesicle Isolation Kit (Takara Bio) | Spin column | [[53]](https://paperpile.com/c/33ouGt/4n2K) |
|  | ExoQuick® ULTRA EV Isolation Kit for Serum and Plasma (System Biosciences) | Bipartate resin columns | [[54]](https://paperpile.com/c/33ouGt/FBb1) |
|  | The EasySep™ Human Pan-Extracellular Vesicle Positive Selection Kit (STEMCELL Technologies Inc.) | Immunoassay | [[55]](https://paperpile.com/c/33ouGt/1xGx) |
|  | miRCURY Exosome Kits (Qiagen) | Precipitation | [[56]](https://paperpile.com/c/33ouGt/BjS5) |
| **Plasma** | Total Exosome Isolation Kit (from plasma) (Thermo Fisher Scientific ) | Precipitation-based | [[57]](https://paperpile.com/c/33ouGt/R1Sc) |
|  | exoEasy Maxi Kit (Qiagen) | Spin column | [[58]](https://paperpile.com/c/33ouGt/KdZC) |
|  | ExoQuantTM overall exosome capture and quantification assay kit (BioVision Inc.) | Immunoassay | [[59]](https://paperpile.com/c/33ouGt/GtSi) |
|  | Capturem Extracellular Vesicle Isolation Kit (Takara Bio) | Spin column | [[60]](https://paperpile.com/c/33ouGt/qDMl) |
|  | ExoQuick® ULTRA EV Isolation Kit for Serum and Plasma (System Biosciences) | Bipartate resin columns | [[61]](https://paperpile.com/c/33ouGt/OVsZ) |
|  | The Exosome Isolation Kit Pan (Miltenyi Biotec) | Immunoassay | [[62]](https://paperpile.com/c/33ouGt/OdZo) |
|  | The EasySep™ Human Pan-Extracellular Vesicle Positive Selection Kit (STEMCELL Technologies Inc.) | Immunoassay | [[63]](https://paperpile.com/c/33ouGt/NKcr) |
|  | ExoFACS™ (BioVision) | Immunoassay | [[64]](https://paperpile.com/c/33ouGt/mD7h) |
|  | miRCURY Exosome Kits (Qiagen) | Precipitation | [[65]](https://paperpile.com/c/33ouGt/trKp) |
| **Urine** | ExoQuantTM overall exosome capture and quantification assay kit (BioVision Inc.) | Immunoassay | [[59]](https://paperpile.com/c/33ouGt/GtSi) |
|  | Capturem Extracellular Vesicle Isolation Kit (Takara Bio) | Spin column | [[66]](https://paperpile.com/c/33ouGt/Pb3x) |
|  | Exo-Urine EV Isolation Kit (System Biosciences) | Size Exclusion Chromatography | [[67]](https://paperpile.com/c/33ouGt/XCEs) |
|  | The Exosome Isolation Kit Pan (Miltenyi Biotec) | Immunoassay | [[62]](https://paperpile.com/c/33ouGt/OdZo) |
|  | miRCURY Exosome Kits (Qiagen) | Precipitation | [[68]](https://paperpile.com/c/33ouGt/Lggr) |
|  | Ultracentrifugation | - | [[69]](https://paperpile.com/c/33ouGt/MZhj) |
|  | Filtration | - | [[69]](https://paperpile.com/c/33ouGt/MZhj) |
|  | Precipitation | - | [[69]](https://paperpile.com/c/33ouGt/MZhj) |
|  | Affinity Purification | - | [[69]](https://paperpile.com/c/33ouGt/MZhj) |
|  | Microfluidics | - | [[69]](https://paperpile.com/c/33ouGt/MZhj) |
| **Saliva** | ExoFACS™ (BioVision) | Immunoassay | [[70]](https://paperpile.com/c/33ouGt/6gY2) |
|  | Minute™ (Invent Biotechnologies) | SDS-PAGE | [[70]](https://paperpile.com/c/33ouGt/6gY2) |
|  | Exo-spinTM (Cell Guidance Systems) | Size Exclusion Chromatography | [[70]](https://paperpile.com/c/33ouGt/6gY2) |
|  | Saliva Exosome Purification Kit (Norgen) | Resin based separation | [[70]](https://paperpile.com/c/33ouGt/6gY2) |
|  | ExoQuantTM overall exosome capture and quantification assay kit(BioVision Inc.) | Immunoassay | [[59]](https://paperpile.com/c/33ouGt/GtSi) |
|  | Capturem Extracellular Vesicle Isolation Kit | Spin column | [[71]](https://paperpile.com/c/33ouGt/AxOS) |
|  | Immuno-affinity | Immunoassay | [[72]](https://paperpile.com/c/33ouGt/yRUH) |
|  | Ciliated micropillars | Porous silicon nanowire-coated micropillars | [[73]](https://paperpile.com/c/33ouGt/dUms) |
|  | PMMA-based membrane filters | Porous polymer monoliths filtration | [[74]](https://paperpile.com/c/33ouGt/Z8Dp) |
|  | Functionalized surfaces (Exochip) | Immunoassay | [[75]](https://paperpile.com/c/33ouGt/wM4Y) |
| **Pleural effusions** | ExoLution Plus Isolation Kit (Exosome Diagnostics) | Precipitation-based | [[76]](https://paperpile.com/c/33ouGt/n5I6) |
| **Breast milk** | Capturem Extracellular Vesicle Isolation Kit (Takara Bio) | Spin column | [[77]](https://paperpile.com/c/33ouGt/HsQG) |
| **Cerebrospinal fluid** | Capturem Extracellular Vesicle Isolation Kit (Takara Bio) | Spin column | [[78]](https://paperpile.com/c/33ouGt/RuBP) |
|  | miRCURY Exosome Kits (Qiagen) | Precipitation | [[79]](https://paperpile.com/c/33ouGt/sApE) |

**Table 5. The list of methods developed for the isolation of cfNAs from different body fluids.** In the table there are summarized different procedures developed for the extraction of cfNAs from various body fluids.They are sorted based on the recommended source for which were developed and also on the source from which the cfNAs were isolated using a specific kit.

NA - not available.

| **Source** | **Type of cfNAs** | **Kit name (Manufacturer)** | **Type** | **References** |
| --- | --- | --- | --- | --- |
| **Serum** | cfDNA | High Pure Viral Nucleic Acid Large Volume Kit (Roche Applied Science) | Spin column | [[80]](https://paperpile.com/c/33ouGt/bKdL) |
|  |  | Epi proColon 2.0 (Epigenomics AG) | NA | [[80]](https://paperpile.com/c/33ouGt/bKdL) |
|  |  | QIAamp Circulating Nucleic Acid Kit D2 (Qiagen) | Silica membrane column | [[81]](https://paperpile.com/c/33ouGt/rNgt) |
|  |  | QIAamp DNA Blood Mini Kit (Qiagen) | Silica membrane column | [[81]](https://paperpile.com/c/33ouGt/rNgt) |
|  |  | QIAsymphony DSP Circulating DNA Kit (Qiagen) | Magnetic beads | [[81]](https://paperpile.com/c/33ouGt/rNgt) |
|  |  | QIAamp MinElute ccfDNA Kit (Qiagen) | Magnetic beads | [[81]](https://paperpile.com/c/33ouGt/rNgt) |
|  |  | QIAamp DSP virus Kit (Qiagen) | Silica membrane column | [[82]](https://paperpile.com/c/33ouGt/v6bq) |
|  |  | QIAamp UltraSens Virus Kit (Qiagen) | Silica membrane column | [[83]](https://paperpile.com/c/33ouGt/3k6G) |
|  |  | MagMax Cell-Free DNA Isolation Kit (Applied Biosystems) | Magnetic beads | [[81]](https://paperpile.com/c/33ouGt/rNgt) |
|  |  | Maxwell RSC ccDNA Plasma Kit (Promega) | Magnetic beads | [[81]](https://paperpile.com/c/33ouGt/rNgt) |
|  |  | Chemagic Next Prep cfDNA (Perkin Elmer) | Magnetic beads | [[81]](https://paperpile.com/c/33ouGt/rNgt) |
|  |  | NextPrep-Mag cfDNA Isolation Kit (Perkin Elmer) | Magnetic beads | [[81]](https://paperpile.com/c/33ouGt/rNgt) |
|  |  | Nucleospin Plasma XS Kit (Macherey-Nagel) | Silica membrane column | [[81]](https://paperpile.com/c/33ouGt/rNgt) |
|  |  | FitAmp Plasma/Serum DNA Isolation Kit (EpiGentek) | Silica membrane column | [[81]](https://paperpile.com/c/33ouGt/rNgt) |
|  |  | GenElute Blood Genomic DNA kit (Sigma) | Silica membrane column | [[81]](https://paperpile.com/c/33ouGt/rNgt) |
|  |  | Plasma/Serum Cell-Free Circulating DNA Purification Kit (Norgen) | Silica membrane column | [[81]](https://paperpile.com/c/33ouGt/rNgt) |
|  |  | Quick-cfDNA/cfRNA Serum and Plasma Kit (Zymo Research) | Silica membrane column | [[81]](https://paperpile.com/c/33ouGt/rNgt) |
|  |  | InviMag® Free Circulating DNA Kit/ IG | Magnetic beads | [[84]](https://paperpile.com/c/33ouGt/sLla) |
|  |  | PME free-circulating DNA Extraction Kit (Analytik Jena) | Spin column | [[85–88]](https://paperpile.com/c/33ouGt/szsd+zTkS+arxl+dGz2) |
|  |  | Quick-cfDNA Serum & Plasma Kit (Zymo Research) | Combination of chemical and enzymatic methods | [[89]](https://paperpile.com/c/33ouGt/De6b) |
|  |  | Sherlock AX (AA Biotechnology) | Spin column | [[90]](https://paperpile.com/c/33ouGt/OHqq) |
|  |  | ZR serum DNA Kit (Zymo Research) | Spin column | [[91]](https://paperpile.com/c/33ouGt/sdvq) |
|  |  | Phenol-chloroform-based method | Laboratory developed | [[91,92]](https://paperpile.com/c/33ouGt/sdvq+vm8P) |
|  |  | Triton/Heat/Phenol-based method | Laboratory developed | [[93]](https://paperpile.com/c/33ouGt/tGeN) |
|  |  | cfPure™ cell-free DNA extraction kit (Amsbio) | Magnetic beads | [[70]](https://paperpile.com/c/33ouGt/6gY2) |
|  | cfRNA | MagMAX Cell-Free DNA Isolation Kit (Thermo Fisher Scientific) | Magnetic beads | [[94]](https://paperpile.com/c/33ouGt/6JVn) |
|  |  | MagMAX Cell-Free Total Nucleic Acid Isolation Kit (Thermo Fisher Scientific) | Magnetic beads | [[95]](https://paperpile.com/c/33ouGt/NgE6) |
|  |  | Plasma/Serum Circulating and Exosomal RNA Purification Kit (Norgen) | Silica membrane column | [[96]](https://paperpile.com/c/33ouGt/0hdT) |
|  |  | miRNeasy Serum/Plasma Kit (Qiagen) | Silica membrane column | [[97]](https://paperpile.com/c/33ouGt/cx9S) |
| **Plasma** | cfDNA | High Pure Viral Nucleic Acid Large Volume Kit (Roche Applied Science) | Spin column | [[80]](https://paperpile.com/c/33ouGt/bKdL) |
|  |  | InviMag® Free Circulating DNA Kit/ IG | Magnetic beads | [[80]](https://paperpile.com/c/33ouGt/bKdL) |
|  |  | Quick-cfDNA™ Serum & Plasma Kit (Zymo Research) | Silica membrane column | [[80]](https://paperpile.com/c/33ouGt/bKdL) |
|  |  | InviGenius PLUS (STRATEC Biomedical AG) | Magnetic beads | [[80]](https://paperpile.com/c/33ouGt/bKdL) |
|  |  | Plasma/Serum Cell-Free Circulating DNA Purification Midi Kit (Norgen Biotek Corp. ) | Spin column | [[85]](https://paperpile.com/c/33ouGt/szsd) |
|  |  | Maxwell RSC ccfDNA Plasma Kit (Promega) | Magnetic beads | [[85,89]](https://paperpile.com/c/33ouGt/szsd+De6b) |
|  |  | Circulating Cell Free DNA Kit (NeoGeneStar) | Magnetic beads | [[85]](https://paperpile.com/c/33ouGt/szsd) |
|  |  | QIAamp circulating Nucleic Acids Kit (Qiagen) | Silica membrane column | [[83,85–90,92,98–101]](https://paperpile.com/c/33ouGt/szsd+Duns+zTkS+arxl+AheV+n9zb+joHL+vm8P+OHqq+dGz2+3k6G+De6b) |
|  |  | QIAamp DNA Blood Mini Kit (Qiagen) | Silica membrane column | [[83,90,91,99,101–103]](https://paperpile.com/c/33ouGt/AheV+mHj2+joHL+sdvq+OHqq+3k6G+GsUB) |
|  |  | NucleoSpin Plasma XS Kit (Macherey-Nagel) | Silica membrane column | [[92,99,101]](https://paperpile.com/c/33ouGt/AheV+joHL+vm8P) |
|  |  | FitAmp Plasma/Serum DNA Isolation Kit (EpiGentek) | Silica membrane column | [[99,101]](https://paperpile.com/c/33ouGt/AheV+joHL) |
|  |  | Plasma/Serum Cell-Free Circulating DNA Purification Midi Kit (Norgen Biotek Corp) | Spin column | [[85,92]](https://paperpile.com/c/33ouGt/szsd+vm8P) |
|  |  | Genomic Mini AX Body Fluids (AA Biotechnology) | Spin column | [[90]](https://paperpile.com/c/33ouGt/OHqq) |
|  |  | NucleoSpin PlasmaF Kit (Macherey-Nagel) | Spin column | [[102]](https://paperpile.com/c/33ouGt/mHj2) |
|  |  | QIAamp DNA Mini Kit (Qiagen) | Silica membrane column | [[104]](https://paperpile.com/c/33ouGt/IHz0) |
|  |  | QIAamp DSP virus Kit (Qiagen) | Silica membrane column | [[82]](https://paperpile.com/c/33ouGt/v6bq) |
|  |  | QIAamp UltraSens Virus Kit (Qiagen) | Silica membrane column | [[83]](https://paperpile.com/c/33ouGt/3k6G) |
|  |  | QIAamp Virus Spin Kit (Qiagen) | Silica membrane column | [[103]](https://paperpile.com/c/33ouGt/GsUB) |
|  |  | Quick-cfDNA Serum & Plasma Kit (Zymo Research) | Combination of chemical and enzymatic methods | [[88,89]](https://paperpile.com/c/33ouGt/dGz2+De6b) |
|  |  | Plasma/Serum Cell-Free Circulating DNA Purification Mini Kit (Norgen) | Silica membrane column | [[92]](https://paperpile.com/c/33ouGt/vm8P) |
|  |  | Sherlock AX (AA Biotechnology) | Spin column | [[90]](https://paperpile.com/c/33ouGt/OHqq) |
|  |  | ZR serum DNA Kit (Zymo Research) | Spin column | [[91]](https://paperpile.com/c/33ouGt/sdvq) |
|  |  | Maxwell RSC ccfDNA Plasma Kit (Promega) | Magnetic beads | [[85,86,98,100,104]](https://paperpile.com/c/33ouGt/szsd+IHz0+Duns+zTkS+n9zb) |
|  |  | ChargeSwitch gDNA Serum Kit, 0.2-1 mL (Invitrogen) | Magnetic beads | [[91,103]](https://paperpile.com/c/33ouGt/sdvq+GsUB) |
|  |  | Agencourt Genfind Blood and Serum Genomic DNA Isolation Kit (Agencourt Bioscience Corporation) | Magnetic beads | [[103]](https://paperpile.com/c/33ouGt/GsUB) |
|  |  | Chemagic NA Extraction Kit (Perkin-Elmer) | Magnetic beads | [[92]](https://paperpile.com/c/33ouGt/vm8P) |
|  |  | COBAS AmpliPrep Total Nucleic Acid Isolation Kit (Roche) | Magnetic beads | [[82]](https://paperpile.com/c/33ouGt/v6bq) |
|  |  | QIAsymphony Circulating NA Kit (Qiagen) | Magnetic beads | [[88]](https://paperpile.com/c/33ouGt/dGz2) |
|  |  | QIAsymphony DSP Virus/ Pathogen Midi Kit (Qiagen) | Magnetic beads | [[83,87]](https://paperpile.com/c/33ouGt/arxl+3k6G) |
|  |  | EpiQuick Circulating Cell- Free DNA Isolation Kit (EpiGentek) | Magnetic beads | [[86]](https://paperpile.com/c/33ouGt/zTkS) |
|  |  | Mag-Bind Circulating DNA (Omega Bio-Tek) | Magnetic beads | [[85]](https://paperpile.com/c/33ouGt/szsd) |
|  |  | MagMAX Cell-free DNA Isolation Kit (Life Technologies) | Magnetic beads | [[85]](https://paperpile.com/c/33ouGt/szsd) |
|  |  | MagNA Pure LC DNA Isolation Kit - Large Volume (Roche) | Magnetic beads | [[102]](https://paperpile.com/c/33ouGt/mHj2) |
|  |  | MagNA Pure Compact Nucleic Acid Isolation Kit I (Roche) | Magnetic beads | [[88,98]](https://paperpile.com/c/33ouGt/Duns+dGz2) |
|  |  | NEXTprep-Mag cfDNA Isolation Kit (Bioo Scientific) | Magnetic beads | [[86]](https://paperpile.com/c/33ouGt/zTkS) |
|  |  | Circulating Cell Free DNA Kit (NeoGeneStar) | Magnetic beads | [[85]](https://paperpile.com/c/33ouGt/szsd) |
|  |  | Phenol-chloroform | Laboratory developed | [[91,92]](https://paperpile.com/c/33ouGt/sdvq+vm8P) |
|  |  | Sodium iodide method | Laboratory developed | [[91]](https://paperpile.com/c/33ouGt/sdvq) |
|  |  | Guanidine-resin method | Laboratory developed | [[91]](https://paperpile.com/c/33ouGt/sdvq) |
|  |  | Puregene DNA purification System Cell and Tissue Kit (Gentra) | Precipitation | [[91]](https://paperpile.com/c/33ouGt/sdvq) |
|  |  | PME free-circulating DNA Extraction Kit (Analytik Jena) | Spin column | [[85–88]](https://paperpile.com/c/33ouGt/szsd+zTkS+arxl+dGz2) |
|  |  | QIAamp MinElute ccfDNA Kit (Qiagen) | Magnetic beads | [[105]](https://paperpile.com/c/33ouGt/MDCQ) |
|  |  | EZ1 ccfDNA Kits (Qiagen) | Magnetic beads | [[105]](https://paperpile.com/c/33ouGt/MDCQ) |
|  |  | QIAsymphony PAXgene Blood ccfDNA Kit (Qiagen) | Magnetic beads | [[105]](https://paperpile.com/c/33ouGt/MDCQ) |
|  | cfRNA | miRNeasy Serum/Plasma Kit (Qiagen) | Silica membrane column | [[106]](https://paperpile.com/c/33ouGt/rUMK) |
|  |  | miRNeasy 96 Advanced QIAcube HT Kit (Qiagen) | Silica membrane column | [[107]](https://paperpile.com/c/33ouGt/pUYH) |
|  |  | QIAamp Circulating Nucleic Acid Kit D2 (Qiagen) | Silica membrane column | [[81]](https://paperpile.com/c/33ouGt/rNgt) |
|  |  | Plasma/Serum Circulating and Exosomal RNA Purification Kit (Norgen) | Silica membrane column | [[108]](https://paperpile.com/c/33ouGt/xkxT) |
| **Urine** | cfDNA | poly-Lys-coated silica particles | Silica membrane column | [[109]](https://paperpile.com/c/33ouGt/V4L2) |
|  |  | Triamine-modified silica particles | Silica membrane column | [[110]](https://paperpile.com/c/33ouGt/RqRD) |
|  |  | Hybridization capture | Laboratory developed | [[111]](https://paperpile.com/c/33ouGt/gXbO) |
|  |  | Wizard Resin/Guanidinium Thiocyanate | Spin column | [[111]](https://paperpile.com/c/33ouGt/gXbO) |
|  |  | Q Sepharose Anion Exchange Resin | Chromatography-based | [[111]](https://paperpile.com/c/33ouGt/gXbO) |
|  |  | Urine Cell-Free Circulating DNA Purification Mini Kit (Norgen) | Spin column | [[111,112]](https://paperpile.com/c/33ouGt/gXbO+p8Hx) |
|  |  | QIAamp Circulating Nucleic Acid Kit(Qiagen) | Silica membrane column | [[111]](https://paperpile.com/c/33ouGt/gXbO) |
|  |  | MagMAX Cell-Free DNA Isolation Kit (Thermo Fisher Scientific ) | Magnetic beads | [[111,112]](https://paperpile.com/c/33ouGt/gXbO+p8Hx) |
|  |  | NEXTprep-Mag Urine cfDNA Isolation Kit (PerkinElmer) | Magnetic beads | [[113]](https://paperpile.com/c/33ouGt/rAZo) |
|  |  | Urine Cell-Free Circulating DNA Purification Midi Kit(Norgen Biotek) | Silica membrane column | [[113]](https://paperpile.com/c/33ouGt/rAZo) |
|  |  | Quick-DNA Urine Kit (ZymoResearch) | Spin column | [[114,115]](https://paperpile.com/c/33ouGt/LIO3+a758) |
|  |  | QIAsymphony DSP Circulating DNA Kit (Qiagen) | Magnetic beads | [[116]](https://paperpile.com/c/33ouGt/qVwe) |
|  |  | PME free-circulating DNA Extraction Kit (Analytik Jena) | Polymer mediated enrichment followed by centrifugation | [[85–88]](https://paperpile.com/c/33ouGt/szsd+zTkS+arxl+dGz2) |
|  | cfRNA | Urine Cell-Free Circulating RNA Purification Mini Kit | Spin column | [[115]](https://paperpile.com/c/33ouGt/a758) |
|  |  | MicroMini Kit (Qiagen) | NA | [[117]](https://paperpile.com/c/33ouGt/LkRP) |
|  |  | QIAamp Circulating Nucleic Acid Kit D2 (QIAgen) | Silica membrane column | [[81]](https://paperpile.com/c/33ouGt/rNgt) |
| **Saliva** | cfDNA | GeneFixTM Saliva DNA isolation kit (Cell projects) | Spin column | [[70]](https://paperpile.com/c/33ouGt/6gY2) |
|  |  | Oragene®-DNA (DNA Genotek) | Ethanol precipitation | [[70]](https://paperpile.com/c/33ouGt/6gY2) |
|  |  | Saliva DNA Isolation Kit (Norgen ) | Spin column | [[70]](https://paperpile.com/c/33ouGt/6gY2) |
|  |  | Saliva DNA Isolation Kit (BioChain Institute) | Spin column | [[70]](https://paperpile.com/c/33ouGt/6gY2) |
|  |  | Mini·SAL™ Saliva DNA Isolation Kit (Oasis Diagnostics® Corporation) | Spin column | [[70]](https://paperpile.com/c/33ouGt/6gY2) |
|  |  | Sherlock AX (AA Biotechnology) | Spin column | [[90]](https://paperpile.com/c/33ouGt/OHqq) |
|  |  | Quick-cfDNA Serum & Plasma Kit (Zymo Research) | Combination of chemical and enzymatic methods | [[118]](https://paperpile.com/c/33ouGt/ZeW7) |
|  |  | Chitosan coated PMMA high surface area | Functionalized surface | [[119]](https://paperpile.com/c/33ouGt/wf7D) |
|  |  | Digital PCR using droplet based microfluidics | Droplet based microfluidics | [[120]](https://paperpile.com/c/33ouGt/58mR) |
|  |  | Tagmentation chemistry and solid phase reversible immobilization (SPRI) based integrated microfluidics | Tagmentation chemistry: Extraction DNA  SPRI: Purification DNA | [[121]](https://paperpile.com/c/33ouGt/diea) |
|  |  | DNA purification and PCR amplification based integrated microfluidics | Micro-sample processing device (μSPD)  DNA purification process | [[122]](https://paperpile.com/c/33ouGt/UKYL) |
|  |  | DNA extraction, amplification, detection based integrated microfluidics | Monolithic aluminum oxide membrane for DNA extraction: seven parallel reaction wells | [[123]](https://paperpile.com/c/33ouGt/UTRg) |
|  | cfRNA | mirVana Isolation Kit (Thermo Fisher Scientific) | Organic extraction followed by purification on a GFF under specialized binding and wash conditions. | [[124]](https://paperpile.com/c/33ouGt/LOqt) |
| **Stool** | cfDNA | QIAamp Circulating Nucleic Acid Kit (Qiagen) | Silica membrane column | [[125]](https://paperpile.com/c/33ouGt/TZep) |
|  |  | NucleoSpin® Gel and PCR Clean-up kit (Macherey-Nagel) | Spin column | [[125]](https://paperpile.com/c/33ouGt/TZep) |
|  |  | NucleoSpin® Plasma XS kit (Macherey-Nagel) | Spin column | [[125]](https://paperpile.com/c/33ouGt/TZep) |
|  |  | cfPure® Cell-Free DNA Extraction Kit (BioChain) | Magnetic beads | [[125]](https://paperpile.com/c/33ouGt/TZep) |
|  |  | MagMAX™ Cell-Free DNA Isolation Kit (Thermo Fisher Scientific) | Magnetic beads | [[125]](https://paperpile.com/c/33ouGt/TZep) |
|  |  | MagNA Pure 24 System (Roche) | Magnetic beads | [[125]](https://paperpile.com/c/33ouGt/TZep) |
| **Seminal plasma** | cfDNA | QIAamp Circulating Nucleic Acid Kit (Qiagen) | Silica membrane column | [[4]](https://paperpile.com/c/33ouGt/XxSj) |
|  |  | QIAamp DNA Mini Kit (Qiagen) | Silica membrane column | [[126]](https://paperpile.com/c/33ouGt/iGgR) |
| **Pleural effusions** | cfDNA | High Pure PCR Template Preparation Kit (Roche) | Spin column | [[127]](https://paperpile.com/c/33ouGt/WaGH) |
|  |  | QIAsymphony DSP DNA Midi Kit (Qiagen) | Silica membrane column | [[128]](https://paperpile.com/c/33ouGt/VA5t) |
|  |  | QIAamp Circulating Nucleic Acid Kit (Qiagen) | Silica membrane column | [[76]](https://paperpile.com/c/33ouGt/n5I6) |
| **Breast milk** | cfDNA | cfDNA purification kit (SummerBio) | NA | [[28]](https://paperpile.com/c/33ouGt/K4Oa) |
|  | cfRNA | cfRNA purification kit (SummerBio) | NA | [[28]](https://paperpile.com/c/33ouGt/K4Oa) |
| **Cerebrospinal fluid** | cfDNA | Quick-cfDNA Serum & Plasma Kit (Zymo Research) | Combination of chemical and enzymatic methods | [[89]](https://paperpile.com/c/33ouGt/De6b) |
| **Amniotic fluid** | cfDNA | Quick-cfDNA Serum & Plasma Kit (Zymo Research) | Combination of chemical and enzymatic methods | [[89]](https://paperpile.com/c/33ouGt/De6b) |
|  | cfRNA | QIAamp Circulating Nucleic Acid kit (Qiagen) | Silica membrane column | [[129]](https://paperpile.com/c/33ouGt/AdeJ) |

**References**

1. [Park, J.-L.; Kim, H.J.; Choi, B.Y.; Lee, H.-C.; Jang, H.-R.; Song, K.S.; Noh, S.-M.; Kim, S.-Y.; Han, D.S.; Kim, Y.S. Quantitative analysis of cell-free DNA in the plasma of gastric cancer patients. *Oncol. Lett.* **2012**, *3*, 921–926.](http://paperpile.com/b/33ouGt/z5al)

2. [Ding, S.; Song, X.; Geng, X.; Liu, L.; Ma, H.; Wang, X.; Wei, L.; Xie, L.; Song, X. Saliva-derived cfDNA is applicable for EGFR mutation detection but not for quantitation analysis in non-small cell lung cancer. *Thorac Cancer* **2019**, *10*, 1973–1983.](http://paperpile.com/b/33ouGt/0Apc)

3. [Bryzgunova, O.E.; Skvortsova, T.E.; Kolesnikova, E.V.; Starikov, A.V.; Rykova, E.Y.; Vlassov, V.V.; Laktionov, P.P. Isolation and comparative study of cell-free nucleic acids from human urine. *Ann. N. Y. Acad. Sci.* **2006**, *1075*, 334–340.](http://paperpile.com/b/33ouGt/d3Kh)

4. [Ponti, G.; Maccaferri, M.; Mandrioli, M.; Manfredini, M.; Micali, S.; Cotugno, M.; Bianchi, G.; Ozben, T.; Pellacani, G.; Del Prete, C.; et al. Seminal Cell-Free DNA Assessment as a Novel Prostate Cancer Biomarker. *Pathol. Oncol. Res.* **2018**, *24*, 941–945.](http://paperpile.com/b/33ouGt/XxSj)

5. [Li, H.-G.; Huang, S.-Y.; Zhou, H.; Liao, A.-H.; Xiong, C.-L. Quick recovery and characterization of cell-free DNA in seminal plasma of normozoospermia and azoospermia: implications for non-invasive genetic utilities. *Asian J. Androl.* **2009**, *11*, 703–709.](http://paperpile.com/b/33ouGt/mJZH)

6. [Tibrewal, S.; Sarkar, J.; Jassim, S.H.; Gandhi, S.; Sonawane, S.; Chaudhary, S.; Byun, Y.-S.; Ivanir, Y.; Hallak, J.; Horner, J.H.; et al. Tear fluid extracellular DNA: diagnostic and therapeutic implications in dry eye disease. *Invest. Ophthalmol. Vis. Sci.* **2013**, *54*, 8051–8061.](http://paperpile.com/b/33ouGt/m9Sw)

7. [Quinones, I.; Daniel, B. Cell free DNA as a component of forensic evidence recovered from touched surfaces. *Forensic Sci. Int. Genet.* **2012**, *6*, 26–30.](http://paperpile.com/b/33ouGt/gP1d)

8. [van der Drift, M.A.; Prinsen, C.F.M.; Hol, B.E.A.; Bolijn, A.S.; Jeunink, M.A.F.; Dekhuijzen, P.N.R.; Thunnissen, F.B.J.M. Can free DNA be detected in sputum of lung cancer patients? *Lung Cancer* **2008**, *61*, 385–390.](http://paperpile.com/b/33ouGt/WKxy)

9. [Husain, H.; Nykin, D.; Bui, N.; Quan, D.; Gomez, G.; Woodward, B.; Venkatapathy, S.; Duttagupta, R.; Fung, E.; Lippman, S.M.; et al. Cell-Free DNA from Ascites and Pleural Effusions: Molecular Insights into Genomic Aberrations and Disease Biology. *Mol. Cancer Ther.* **2017**, *16*, 948–955.](http://paperpile.com/b/33ouGt/QhYO)

10. [Dong, C.; Liu, Y.; Sun, C.; Liang, H.; Dai, L.; Shen, J.; Wei, S.; Guo, S.; Leong, K.W.; Chen, Y.; et al. Identification of Specific Joint-Inflammatogenic Cell-Free DNA Molecules From Synovial Fluids of Patients With Rheumatoid Arthritis. *Front. Immunol.* **2020**, *11*, 662.](http://paperpile.com/b/33ouGt/xV29)

11. [Virzì, G.M.; Milan Manani, S.; Brocca, A.; Cantaluppi, V.; de Cal, M.; Pastori, S.; Tantillo, I.; Zambon, R.; Crepaldi, C.; Ronco, C. Peritoneal Cell-free DNA: an innovative method for determining acute cell damage in peritoneal membrane and for monitoring the recovery process after peritonitis. *J. Nephrol.* **2016**, *29*, 111–118.](http://paperpile.com/b/33ouGt/S1b9)

12. [Pajek, J.; Kveder, R.; Gucek, A.; Skoberne, A.; Bren, A.; Bucar, M.; Cerne, D.; Lukac-Bajalo, J. Cell-free DNA in the peritoneal effluent of peritoneal dialysis solutions. *Ther. Apher. Dial.* **2010**, *14*, 20–26.](http://paperpile.com/b/33ouGt/jaBY)

13. [Park, S.; Hur, J.Y.; Lee, K.Y.; Lee, J.C.; Rho, J.K.; Shin, S.H.; Choi, C.-M. Assessment of EGFR mutation status using cell-free DNA from bronchoalveolar lavage fluid. *Clin. Chem. Lab. Med.* **2017**, *55*, 1489–1495.](http://paperpile.com/b/33ouGt/CJOa)

14. [Utomo, W.K.; Janmaat, V.T.; Verhaar, A.P.; Cros, J.; Lévy, P.; Ruszniewski, P.; den Berg, M.S.V.; Jenster, G.; Bruno, M.J.; Braat, H.; et al. DNA integrity as biomarker in pancreatic cyst fluid. *Am. J. Cancer Res.* **2016**, *6*, 1837–1841.](http://paperpile.com/b/33ouGt/8VII)

15. [Gerrish, A.; Stone, E.; Clokie, S.; Ainsworth, J.R.; Jenkinson, H.; McCalla, M.; Hitchcott, C.; Colmenero, I.; Allen, S.; Parulekar, M.; et al. Non-invasive diagnosis of retinoblastoma using cell-free DNA from aqueous humour. *Br. J. Ophthalmol.* **2019**, doi:](http://paperpile.com/b/33ouGt/Ls9U)[10.1136/bjophthalmol-2018-313005](http://dx.doi.org/10.1136/bjophthalmol-2018-313005)[.](http://paperpile.com/b/33ouGt/Ls9U)

16. [Berry, J.L.; Xu, L.; Murphree, A.L.; Krishnan, S.; Stachelek, K.; Zolfaghari, E.; McGovern, K.; Lee, T.C.; Carlsson, A.; Kuhn, P.; et al. Potential of Aqueous Humor as a Surrogate Tumor Biopsy for Retinoblastoma. *JAMA Ophthalmol.* **2017**, *135*, 1221–1230.](http://paperpile.com/b/33ouGt/vukL)

17. [von Baumgarten, L.; Kumbrink, J.; Jung, A.; Reischer, A.; Flach, M.; Liebmann, S.; Metzeler, K.H.; Holch, J.W.; Niyazi, M.; Thon, N.; et al. Therapeutic management of neuro-oncologic patients - potential relevance of CSF liquid biopsy. *Theranostics* **2020**, *10*, 856–866.](http://paperpile.com/b/33ouGt/dVV2)

18. [Bianchi, D.W.; LeShane, E.S.; Cowan, J.M. Large amounts of cell-free fetal DNA are present in amniotic fluid. *Clin. Chem.* **2001**, *47*, 1867–1869.](http://paperpile.com/b/33ouGt/TYGx)

19. [Weber, J.A.; Baxter, D.H.; Zhang, S.; Huang, D.Y.; Huang, K.H.; Lee, M.J.; Galas, D.J.; Wang, K. The microRNA spectrum in 12 body fluids. *Clin. Chem.* **2010**, *56*, 1733–1741.](http://paperpile.com/b/33ouGt/nPnE)

20. [Beck, T.N.; Boumber, Y.A.; Aggarwal, C.; Pei, J.; Thrash-Bingham, C.; Fittipaldi, P.; Vlasenkova, R.; Rao, C.; Borghaei, H.; Cristofanilli, M.; et al. Circulating tumor cell and cell-free RNA capture and expression analysis identify platelet-associated genes in metastatic lung cancer. *BMC Cancer* **2019**, *19*, 603.](http://paperpile.com/b/33ouGt/sHSQ)

21. [Huang, S.; Li, H.; Ding, X.; Xiong, C. Presence and characterization of cell-free seminal RNA in healthy individuals: implications for noninvasive disease diagnosis and gene expression studies of the male reproductive system. *Clin. Chem.* **2009**, *55*, 1967–1976.](http://paperpile.com/b/33ouGt/i9Mv)

22. [Spinillo, A.; Debiaggi, M.; Zara, F.; Maserati, R.; Polatti, F.; De Santolo, A. Factors associated with nucleic acids related to human immunodeficiency virus type 1 in cervico-vaginal secretions. *BJOG* **2001**, *108*, 634–641.](http://paperpile.com/b/33ouGt/Ak7S)

23. [Gardella, B.; Roccio, M.; Maccabruni, A.; Mariani, B.; Panzeri, L.; Zara, F.; Spinillo, A. HIV shedding in cervico-vaginal secretions in pregnant women. *Curr. HIV Res.* **2011**, *9*, 313–320.](http://paperpile.com/b/33ouGt/RJ4f)

24. [Parpart-Li, S.; Bartlett, B.; Popoli, M.; Adleff, V.; Tucker, L.; Steinberg, R.; Georgiadis, A.; Phallen, J.; Brahmer, J.; Azad, N.; et al. The Effect of Preservative and Temperature on the Analysis of Circulating Tumor DNA. *Clin. Cancer Res.* **2017**, *23*, 2471–2477.](http://paperpile.com/b/33ouGt/Jeq7)

25. [Wu, Z.; Yang, Z.; Li, C.S.; Zhao, W.; Liang, Z.X.; Dai, Y.; Zhu, Q.; Miao, K.L.; Cui, D.H.; Chen, L.A. Differences in the genomic profiles of cell-free DNA between plasma, sputum, urine, and tumor tissue in advanced NSCLC. *Cancer Med.* **2019**, *8*, 910–919.](http://paperpile.com/b/33ouGt/F1ml)

26. [Su, Y.-H.; Wang, M.; Brenner, D.E.; Ng, A.; Melkonyan, H.; Umansky, S.; Syngal, S.; Block, T.M. Human urine contains small, 150 to 250 nucleotide-sized, soluble DNA derived from the circulation and may be useful in the detection of colorectal cancer. *J. Mol. Diagn.* **2004**, *6*, 101–107.](http://paperpile.com/b/33ouGt/yOOl)

27. [Shen, N.; Zhang, D.; Yin, L.; Qiu, Y.; Liu, J.; Yu, W.; Fu, X.; Zhu, B.; Xu, X.; Duan, A.; et al. Bile cell‑free DNA as a novel and powerful liquid biopsy for detecting somatic variants in biliary tract cancer. *Oncol. Rep.* **2019**, *42*, 549–560.](http://paperpile.com/b/33ouGt/MoiH)

28. [Song, Q.; Zhang, Y.; Liu, H.; Du, Y. Potential of Using Cell-Free DNA and miRNA in Breast Milk to Screen Early Breast Cancer. *Biomed Res. Int.* **2020**, *2020*, doi:](http://paperpile.com/b/33ouGt/K4Oa)[10.1155/2020/8126176](http://dx.doi.org/10.1155/2020/8126176)[.](http://paperpile.com/b/33ouGt/K4Oa)

29. [Tsui, N.B.Y.; Jiang, P.; Chow, K.C.K.; Su, X.; Leung, T.Y.; Sun, H.; Chan, K.C.A.; Chiu, R.W.K.; Lo, Y.M.D. High resolution size analysis of fetal DNA in the urine of pregnant women by paired-end massively parallel sequencing. *PLoS One* **2012**, *7*, e48319.](http://paperpile.com/b/33ouGt/Q0IH)

30. [marcher Stabilization Available online:](http://paperpile.com/b/33ouGt/23Vt) <https://www.streck.com/products/stabilization/> [(accessed on Sep 6, 2020).](http://paperpile.com/b/33ouGt/23Vt)

31. [CELLSEARCH® System Overview Available online:](http://paperpile.com/b/33ouGt/aRRr) <https://www.cellsearchctc.com/product-systems-overview/cellsearch-system-overview> [(accessed on Sep 6, 2020).](http://paperpile.com/b/33ouGt/aRRr)

32. [PAXgene Blood ccfDNA Tubes (100) Available online:](http://paperpile.com/b/33ouGt/Xtg6) <https://www.qiagen.com/us/products/discovery-and-translational-research/sample-collection-stabilization/dna/paxgene-blood-ccfdna-tube/#orderinginformation> [(accessed on Sep 6, 2020).](http://paperpile.com/b/33ouGt/Xtg6)

33. [preanalytix.com: PAXgene Blood ccfDNA Tube (CE-IVD) Available online:](http://paperpile.com/b/33ouGt/L2i8) <https://www.preanalytix.com/products/blood/ccfdna/paxgene-blood-ccfdna-tube-ce-ivd/SK?cHash=40383c5a4307b4dd75054d09b6d55bbb> [(accessed on Sep 6, 2020).](http://paperpile.com/b/33ouGt/L2i8)

34. [Cell-Free DNA Collection Tube Available online:](http://paperpile.com/b/33ouGt/TxyL) <https://sequencing.roche.com/en/products-solutions/products/sample-collection/cell-free-dna-collection-tube.html> [(accessed on Sep 6, 2020).](http://paperpile.com/b/33ouGt/TxyL)

35. [cf-DNA/cf-RNA Preservative Tubes (Cat. 63950) Available online:](http://paperpile.com/b/33ouGt/O3os) <https://norgenbiotek.com/product/cf-dnacf-rna-preservative-tubes> [(accessed on Sep 6, 2020).](http://paperpile.com/b/33ouGt/O3os)

36. [LBgard® Blood Tubes Available online:](http://paperpile.com/b/33ouGt/7uo5) <https://www.biomatrica.com/product/lbgard-blood-tubes/> [(accessed on Sep 6, 2020).](http://paperpile.com/b/33ouGt/7uo5)

37. [Cfgenome, LLC v. Streck, Inc., 4:16CV3130 Available online:](http://paperpile.com/b/33ouGt/51WQ) <https://casetext.com/case/cfgenome-llc-v-streck-inc-1> [(accessed on Sep 6, 2020).](http://paperpile.com/b/33ouGt/51WQ)

38. [Blood STASIS^TM^ 21-cfDNA Blood Collection Tubes Available online:](http://paperpile.com/b/33ouGt/NEIz) <https://www.magbiogenomics.com/blood-stasistm-21-cfdna-blood-collection-tubes.html> [(accessed on Sep 6, 2020).](http://paperpile.com/b/33ouGt/NEIz)

39. [Website Available online:](http://paperpile.com/b/33ouGt/BD7f) <https://biocept.com/cee-sure/> [(accessed on Sep 6, 2020).](http://paperpile.com/b/33ouGt/BD7f)

40. [Website Available online:](http://paperpile.com/b/33ouGt/wsML) <https://www.inresearchmed.com/improgene-cell-free-dna-tube> [(accessed on Sep 6, 2020).](http://paperpile.com/b/33ouGt/wsML)

41. [NICE® Check cfDNA Tube Available online:](http://paperpile.com/b/33ouGt/ZWlb) <https://www.omnia-health.com/product/nice%C2%AE-check-cfdna-tube> [(accessed on Sep 6, 2020).](http://paperpile.com/b/33ouGt/ZWlb)

42. [Saliva Exosome Collection and Preservation Kit (Cat. 65400) Available online:](http://paperpile.com/b/33ouGt/aIUn) <https://norgenbiotek.com/product/saliva-exosome-collection-and-preservation-kit> [(accessed on Sep 8, 2020).](http://paperpile.com/b/33ouGt/aIUn)

43. [Home Page - Oasis Diagnostics® Available online:](http://paperpile.com/b/33ouGt/gDXV) <https://4saliva.com/> [(accessed on Sep 6, 2020).](http://paperpile.com/b/33ouGt/gDXV)

44. [marcher Cell-Free DNA Urine Preserve Available online:](http://paperpile.com/b/33ouGt/gsxi) <https://www.streck.com/products/stabilization/cell-free-dna-urine-preserve/> [(accessed on Sep 6, 2020).](http://paperpile.com/b/33ouGt/gsxi)

45. [Urine Conditioning Buffer Available online:](http://paperpile.com/b/33ouGt/AoRo) <https://www.zymoresearch.com/products/urine-conditioning-buffer> [(accessed on Sep 6, 2020).](http://paperpile.com/b/33ouGt/AoRo)

46. [Urine Preservation (Cat. 18116, 18118, 18120) Available online:](http://paperpile.com/b/33ouGt/wAv7) <https://norgenbiotek.com/product/urine-preservation> [(accessed on Sep 6, 2020).](http://paperpile.com/b/33ouGt/wAv7)

47. [DNA Genotek - Saliva Microbiome DNA/RNA Collection Kit Available online:](http://paperpile.com/b/33ouGt/dMKw) <https://www.dnagenotek.com/ROW/products/collection-infectious-disease/colli-pee/FV-5000.html#n1> [(accessed on Sep 6, 2020).](http://paperpile.com/b/33ouGt/dMKw)

48. [DNA/RNA Shield Fecal Collection Tube Available online:](http://paperpile.com/b/33ouGt/E4JG) <https://www.zymoresearch.com/products/dna-rna-shield-fecal-collection-tube> [(accessed on Sep 6, 2020).](http://paperpile.com/b/33ouGt/E4JG)

49. [Stool Sample Preparation Devices Available online:](http://paperpile.com/b/33ouGt/b8iO) <https://norgenbiotek.com/category/stool-sample-collection-and-preservation-devices> [(accessed on Sep 6, 2020).](http://paperpile.com/b/33ouGt/b8iO)

50. [DNA Genotek - Gut Microbiome DNA Collection Kit from Stool/Feces Available online:](http://paperpile.com/b/33ouGt/5Vga) <https://www.dnagenotek.com/ROW/products/collection-microbiome/omnigene-gut/OM-200.html> [(accessed on Sep 6, 2020).](http://paperpile.com/b/33ouGt/5Vga)

51. [Trusted exosome purification Available online:](http://paperpile.com/b/33ouGt/h0Dx) <https://www.cellgs.com/products/exo-spinand8482.html> [(accessed on Sep 6, 2020).](http://paperpile.com/b/33ouGt/h0Dx)

52. [exoEasy Maxi Kit Available online:](http://paperpile.com/b/33ouGt/Mb2P) <https://www.qiagen.com/ch/products/discovery-and-translational-research/exosomes-ctcs/exosomes/exoeasy-maxi-kit/#orderinginformation> [(accessed on Sep 6, 2020).](http://paperpile.com/b/33ouGt/Mb2P)

53. [Website Available online:](http://paperpile.com/b/33ouGt/4n2K) <http://catalog.takara-bio.co.jp/PDFS/Rapid,-pure,-and-concentrated-purification-of-extracellular-vesicles-from-biofluids.pdf> [(accessed on Sep 6, 2020).](http://paperpile.com/b/33ouGt/4n2K)

54. [The Purest and Highest Yielding EV Isolation System Available online:](http://paperpile.com/b/33ouGt/FBb1) <https://systembio.com/shop/exoquick-ultra-ev-isolation-kit-serum-plasma/> [(accessed on Sep 6, 2020).](http://paperpile.com/b/33ouGt/FBb1)

55. [EasySep^TM^ Human Pan-Extracellular Vesicle Positive Selection Kit Available online:](http://paperpile.com/b/33ouGt/1xGx) <https://www.stemcell.com/easysep-human-pan-extracellular-vesicle-positive-selection-kit.html> [(accessed on Sep 6, 2020).](http://paperpile.com/b/33ouGt/1xGx)

56. [miRCURY Exosome Kits Available online:](http://paperpile.com/b/33ouGt/BjS5) <https://www.qiagen.com/us/products/discovery-and-translational-research/exosomes-ctcs/exosomes/mircury-exosome-kits/#productdetails> [(accessed on Sep 6, 2020).](http://paperpile.com/b/33ouGt/BjS5)

57. [Total Exosome Isolation Kit (from plasma) Available online:](http://paperpile.com/b/33ouGt/R1Sc) <https://www.thermofisher.com/order/catalog/product/4484450#/4484450> [(accessed on Sep 6, 2020).](http://paperpile.com/b/33ouGt/R1Sc)

58. [exoEasy Maxi Kit Available online:](http://paperpile.com/b/33ouGt/KdZC) <https://www.qiagen.com/ch/products/discovery-and-translational-research/exosomes-ctcs/exosomes/exoeasy-maxi-kit/#orderinginformation> [(accessed on Sep 6, 2020).](http://paperpile.com/b/33ouGt/KdZC)

59. [Elsharkawi, F.; Elsabah, M.; Shabayek, M.; Khaled, H. Urine and Serum Exosomes as Novel Biomarkers in Detection of Bladder Cancer. *Asian Pac. J. Cancer Prev.* **2019**, *20*, 2219–2224.](http://paperpile.com/b/33ouGt/GtSi)

60. [[No title] Available online:](http://paperpile.com/b/33ouGt/qDMl) <http://catalog.takara-bio.co.jp/PDFS/Rapid,-pure,-and-concentrated-purification-of-extracellular-vesicles-from-biofluids.pdf> [(accessed on Sep 6, 2020).](http://paperpile.com/b/33ouGt/qDMl)

61. [The Purest and Highest Yielding EV Isolation System Available online:](http://paperpile.com/b/33ouGt/OVsZ) <https://systembio.com/shop/exoquick-ultra-ev-isolation-kit-serum-plasma/> [(accessed on Sep 6, 2020).](http://paperpile.com/b/33ouGt/OVsZ)

62. [Exosome Isolation Kit CD9, human - Exosome isolation - Organelle research - Reagents - MACSmolecular - Products - Miltenyi Biotec - USA Available online:](http://paperpile.com/b/33ouGt/OdZo) <https://www.miltenyibiotec.com/US-en/products/exosome-isolation-kit-cd9-human.html> [(accessed on Sep 6, 2020).](http://paperpile.com/b/33ouGt/OdZo)

63. [EasySep^TM^ Human Pan-Extracellular Vesicle Positive Selection Kit Available online:](http://paperpile.com/b/33ouGt/NKcr) <https://www.stemcell.com/easysep-human-pan-extracellular-vesicle-positive-selection-kit.html> [(accessed on Sep 6, 2020).](http://paperpile.com/b/33ouGt/NKcr)

64. [ExoFACS^TM^ Kit for Plasma Exosomes, BioVision Inc Available online:](http://paperpile.com/b/33ouGt/mD7h) <https://us.vwr.com/store/product/18918055/exofacstm-kit-for-plasma-exosomes-biovision-inc> [(accessed on Sep 6, 2020).](http://paperpile.com/b/33ouGt/mD7h)

65. [miRCURY Exosome Kits Available online:](http://paperpile.com/b/33ouGt/trKp) <https://www.qiagen.com/us/products/discovery-and-translational-research/exosomes-ctcs/exosomes/mircury-exosome-kits/#productdetails> [(accessed on Sep 6, 2020).](http://paperpile.com/b/33ouGt/trKp)

66. [[No title] Available online:](http://paperpile.com/b/33ouGt/Pb3x) <http://catalog.takara-bio.co.jp/PDFS/Rapid,-pure,-and-concentrated-purification-of-extracellular-vesicles-from-biofluids.pdf> [(accessed on Sep 6, 2020).](http://paperpile.com/b/33ouGt/Pb3x)

67. [Extracellular vesicle isolation from urine: Exo-Urine EV Isolation Kit Available online:](http://paperpile.com/b/33ouGt/XCEs) <https://www.bioscience.co.uk/cpl/exo-urine> [(accessed on Sep 6, 2020).](http://paperpile.com/b/33ouGt/XCEs)

68. [miRCURY Exosome Kits Available online:](http://paperpile.com/b/33ouGt/Lggr) <https://www.qiagen.com/us/products/discovery-and-translational-research/exosomes-ctcs/exosomes/mircury-exosome-kits/#productdetails> [(accessed on Sep 6, 2020).](http://paperpile.com/b/33ouGt/Lggr)

69. [Street, J.M.; Koritzinsky, E.H.; Glispie, D.M.; Star, R.A.; Yuen, P.S.T. Urine Exosomes: An Emerging Trove of Biomarkers. *Adv. Clin. Chem.* **2017**, *78*, 103–122.](http://paperpile.com/b/33ouGt/MZhj)

70. [Hyun, K.-A.; Gwak, H.; Lee, J.; Kwak, B.; Jung, H.-I. Salivary Exosome and Cell-Free DNA for Cancer Detection. *Micromachines (Basel)* **2018**, *9*, doi:](http://paperpile.com/b/33ouGt/6gY2)[10.3390/mi9070340](http://dx.doi.org/10.3390/mi9070340)[.](http://paperpile.com/b/33ouGt/6gY2)

71. [[No title] Available online:](http://paperpile.com/b/33ouGt/AxOS) <http://catalog.takara-bio.co.jp/PDFS/Rapid,-pure,-and-concentrated-purification-of-extracellular-vesicles-from-biofluids.pdf> [(accessed on Sep 7, 2020).](http://paperpile.com/b/33ouGt/AxOS)

72. [He, M.; Crow, J.; Roth, M.; Zeng, Y.; Godwin, A.K. Integrated immunoisolation and protein analysis of circulating exosomes using microfluidic technology. *Lab Chip* **2014**, *14*, 3773–3780.](http://paperpile.com/b/33ouGt/yRUH)

73. [Wang, Z.; Wu, H.-J.; Fine, D.; Schmulen, J.; Hu, Y.; Godin, B.; Zhang, J.X.J.; Liu, X. Ciliated micropillars for the microfluidic-based isolation of nanoscale lipid vesicles. *Lab Chip* **2013**, *13*, 2879–2882.](http://paperpile.com/b/33ouGt/dUms)

74. [Davies, R.T.; Kim, J.; Jang, S.C.; Choi, E.-J.; Gho, Y.S.; Park, J. Microfluidic filtration system to isolate extracellular vesicles from blood. *Lab Chip* **2012**, *12*, 5202–5210.](http://paperpile.com/b/33ouGt/Z8Dp)

75. [Kanwar, S.S.; Dunlay, C.J.; Simeone, D.M.; Nagrath, S. Microfluidic device (ExoChip) for on-chip isolation, quantification and characterization of circulating exosomes. *Lab Chip* **2014**, *14*, 1891–1900.](http://paperpile.com/b/33ouGt/wM4Y)

76. [Song, Z.; Cai, Z.; Yan, J.; Shao, Y.W.; Zhang, Y. Liquid biopsies using pleural effusion-derived exosomal DNA in advanced lung adenocarcinoma. *Transl Lung Cancer Res* **2019**, *8*, 392–400.](http://paperpile.com/b/33ouGt/n5I6)

77. [[No title] Available online:](http://paperpile.com/b/33ouGt/HsQG) <http://catalog.takara-bio.co.jp/PDFS/Rapid,-pure,-and-concentrated-purification-of-extracellular-vesicles-from-biofluids.pdf> [(accessed on Sep 7, 2020).](http://paperpile.com/b/33ouGt/HsQG)

78. [[No title] Available online:](http://paperpile.com/b/33ouGt/RuBP) <http://catalog.takara-bio.co.jp/PDFS/Rapid,-pure,-and-concentrated-purification-of-extracellular-vesicles-from-biofluids.pdf> [(accessed on Sep 7, 2020).](http://paperpile.com/b/33ouGt/RuBP)

79. [miRCURY Exosome Kits Available online:](http://paperpile.com/b/33ouGt/sApE) <https://www.qiagen.com/us/products/discovery-and-translational-research/exosomes-ctcs/exosomes/mircury-exosome-kits/#productdetails> [(accessed on Sep 7, 2020).](http://paperpile.com/b/33ouGt/sApE)

80. [Barták, B.K.; Kalmár, A.; Galamb, O.; Wichmann, B.; Nagy, Z.B.; Tulassay, Z.; Dank, M.; Igaz, P.; Molnár, B. Blood Collection and Cell-Free DNA Isolation Methods Influence the Sensitivity of Liquid Biopsy Analysis for Colorectal Cancer Detection. *Pathol. Oncol. Res.* **2019**, *25*, 915–923.](http://paperpile.com/b/33ouGt/bKdL)

81. [Schweizer, M.T.; Gulati, R.; Beightol, M.; Konnick, E.Q.; Cheng, H.H.; Klemfuss, N.; De Sarkar, N.; Yu, E.Y.; Montgomery, R.B.; Nelson, P.S.; et al. Clinical determinants for successful circulating tumor DNA analysis in prostate cancer. *Prostate* **2019**, *79*, 701–708.](http://paperpile.com/b/33ouGt/rNgt)

82. [Ordoñez, E.; Rueda, L.; Cañadas, M.P.; Fuster, C.; Cirigliano, V. Evaluation of sample stability and automated DNA extraction for fetal sex determination using cell-free fetal DNA in maternal plasma. *Biomed Res. Int.* **2013**, *2013*, 195363.](http://paperpile.com/b/33ouGt/v6bq)

83. [Warton, K.; Graham, L.-J.; Yuwono, N.; Samimi, G. Comparison of 4 commercial kits for the extraction of circulating DNA from plasma. *Cancer Genet.* **2018**, *228-229*, 143–150.](http://paperpile.com/b/33ouGt/3k6G)

84. [[No title] Available online:](http://paperpile.com/b/33ouGt/sLla) <https://www.isogen-lifescience.com/uploads/1Z/LK/1ZLK8yYdP-z2BhhmRI7Arw/Liquid_Biopsy..pdf> [(accessed on Sep 6, 2020).](http://paperpile.com/b/33ouGt/sLla)

85. [Markus, H.; Contente-Cuomo, T.; Farooq, M.; Liang, W.S.; Borad, M.J.; Sivakumar, S.; Gollins, S.; Tran, N.L.; Dhruv, H.D.; Berens, M.E.; et al. Evaluation of pre-analytical factors affecting plasma DNA analysis. *Sci. Rep.* **2018**, *8*, 7375.](http://paperpile.com/b/33ouGt/szsd)

86. [Sorber, L.; Zwaenepoel, K.; Deschoolmeester, V.; Roeyen, G.; Lardon, F.; Rolfo, C.; Pauwels, P. A Comparison of Cell-Free DNA Isolation Kits: Isolation and Quantification of Cell-Free DNA in Plasma. *J. Mol. Diagn.* **2017**, *19*, 162–168.](http://paperpile.com/b/33ouGt/zTkS)

87. [Sherwood, J.L.; Corcoran, C.; Brown, H.; Sharpe, A.D.; Musilova, M.; Kohlmann, A. Optimised Pre-Analytical Methods Improve KRAS Mutation Detection in Circulating Tumour DNA (ctDNA) from Patients with Non-Small Cell Lung Cancer (NSCLC). *PLoS One* **2016**, *11*, e0150197.](http://paperpile.com/b/33ouGt/arxl)

88. [van Ginkel, J.H.; van den Broek, D.A.; van Kuik, J.; Linders, D.; de Weger, R.; Willems, S.M.; Huibers, M.M.H. Preanalytical blood sample workup for cell-free DNA analysis using Droplet Digital PCR for future molecular cancer diagnostics. *Cancer Med.* **2017**, *6*, 2297–2307.](http://paperpile.com/b/33ouGt/dGz2)

89. [Leest, P. van der; Boonstra, P.A.; Elst, A.T.; van Kempen, L.C.; Tibbesma, M.; Koopmans, J.; Miedema, A.; Tamminga, M.; Groen, H.J.M.; Reyners, A.K.L.; et al. Comparison of Circulating Cell-Free DNA Extraction Methods for Downstream Analysis in Cancer Patients. *Cancers*  **2020**, *12*, doi:](http://paperpile.com/b/33ouGt/De6b)[10.3390/cancers12051222](http://dx.doi.org/10.3390/cancers12051222)[.](http://paperpile.com/b/33ouGt/De6b)

90. [Mazurek, A.M.; Fiszer-Kierzkowska, A.; Rutkowski, T.; Składowski, K.; Pierzyna, M.; Scieglińska, D.; Woźniak, G.; Głowacki, G.; Kawczyński, R.; Małusecka, E. Optimization of circulating cell-free DNA recovery for KRAS mutation and HPV detection in plasma. *Cancer Biomark.* **2013**, *13*, 385–394.](http://paperpile.com/b/33ouGt/OHqq)

91. [Fong, S.L.; Zhang, J.T.; Lim, C.K.; Eu, K.W.; Liu, Y. Comparison of 7 methods for extracting cell-free DNA from serum samples of colorectal cancer patients. *Clin. Chem.* **2009**, *55*, 587–589.](http://paperpile.com/b/33ouGt/sdvq)

92. [Mauger, F.; Dulary, C.; Daviaud, C.; Deleuze, J.-F.; Tost, J. Comprehensive evaluation of methods to isolate, quantify, and characterize circulating cell-free DNA from small volumes of plasma. *Anal. Bioanal. Chem.* **2015**, *407*, 6873–6878.](http://paperpile.com/b/33ouGt/vm8P)

93. [Xue, X.; Teare, M.D.; Holen, I.; Zhu, Y.M.; Woll, P.J. Optimizing the yield and utility of circulating cell-free DNA from plasma and serum. *Clin. Chim. Acta* **2009**, *404*, 100–104.](http://paperpile.com/b/33ouGt/tGeN)

94. [MagMAX^TM^ Cell-Free DNA Isolation Kit Available online:](http://paperpile.com/b/33ouGt/6JVn) <https://www.thermofisher.com/order/catalog/product/A29319?SID=srch-hj-A29319#/A29319?SID=srch-hj-A29319> [(accessed on Sep 6, 2020).](http://paperpile.com/b/33ouGt/6JVn)

95. [MagMAX^TM^ Cell-Free Total Nucleic Acid Isolation Kit Available online:](http://paperpile.com/b/33ouGt/NgE6) <https://www.thermofisher.com/order/catalog/product/A36716?SID=srch-hj-A36716#/A36716?SID=srch-hj-A36716> [(accessed on Sep 6, 2020).](http://paperpile.com/b/33ouGt/NgE6)

96. [Plasma/Serum Circulating and Exosomal RNA Purification Kit (Slurry Format) (Cat. 42800) Available online:](http://paperpile.com/b/33ouGt/0hdT) <https://norgenbiotek.com/product/plasmaserum-circulating-and-exosomal-rna-purification-kit-slurry-format> [(accessed on Sep 6, 2020).](http://paperpile.com/b/33ouGt/0hdT)

97. [miRNeasy Serum/Plasma Kit Available online:](http://paperpile.com/b/33ouGt/cx9S) <https://www.qiagen.com/us/products/diagnostics-and-clinical-research/sample-processing/mirneasy-serumplasma-kit/#orderinginformation> [(accessed on Sep 6, 2020).](http://paperpile.com/b/33ouGt/cx9S)

98. [Pérez-Barrios, C.; Nieto-Alcolado, I.; Torrente, M.; Jiménez-Sánchez, C.; Calvo, V.; Gutierrez-Sanz, L.; Palka, M.; Donoso-Navarro, E.; Provencio, M.; Romero, A. Comparison of methods for circulating cell-free DNA isolation using blood from cancer patients: impact on biomarker testing. *Transl Lung Cancer Res* **2016**, *5*, 665–672.](http://paperpile.com/b/33ouGt/Duns)

99. [Page, K.; Guttery, D.S.; Zahra, N.; Primrose, L.; Elshaw, S.R.; Pringle, J.H.; Blighe, K.; Marchese, S.D.; Hills, A.; Woodley, L.; et al. Influence of plasma processing on recovery and analysis of circulating nucleic acids. *PLoS One* **2013**, *8*, e77963.](http://paperpile.com/b/33ouGt/AheV)

100. [Kloten, V.; Rüchel, N.; Brüchle, N.O.; Gasthaus, J.; Freudenmacher, N.; Steib, F.; Mijnes, J.; Eschenbruch, J.; Binnebösel, M.; Knüchel, R.; et al. Liquid biopsy in colon cancer: comparison of different circulating DNA extraction systems following absolute quantification of mutations using Intplex allele-specific PCR. *Oncotarget* **2017**, *8*, 86253–86263.](http://paperpile.com/b/33ouGt/n9zb)

101. [Devonshire, A.S.; Whale, A.S.; Gutteridge, A.; Jones, G.; Cowen, S.; Foy, C.A.; Huggett, J.F. Towards standardisation of cell-free DNA measurement in plasma: controls for extraction efficiency, fragment size bias and quantification. *Anal. Bioanal. Chem.* **2014**, *406*, 6499–6512.](http://paperpile.com/b/33ouGt/joHL)

102. [Fleischhacker, M.; Schmidt, B.; Weickmann, S.; Fersching, D.M.I.; Leszinski, G.S.; Siegele, B.; Stötzer, O.J.; Nagel, D.; Holdenrieder, S. Methods for isolation of cell-free plasma DNA strongly affect DNA yield. *Clin. Chim. Acta* **2011**, *412*, 2085–2088.](http://paperpile.com/b/33ouGt/mHj2)

103. [Board, R.E.; Williams, V.S.; Knight, L.; Shaw, J.; Greystoke, A.; Ranson, M.; Dive, C.; Blackhall, F.H.; Hughes, A. Isolation and extraction of circulating tumor DNA from patients with small cell lung cancer. *Ann. N. Y. Acad. Sci.* **2008**, *1137*, 98–107.](http://paperpile.com/b/33ouGt/GsUB)

104. [Sato, A.; Nakashima, C.; Abe, T.; Kato, J.; Hirai, M.; Nakamura, T.; Komiya, K.; Kimura, S.; Sueoka, E.; Sueoka-Aragane, N. Investigation of appropriate pre-analytical procedure for circulating free DNA from liquid biopsy. *Oncotarget* **2018**, *9*, 31904–31914.](http://paperpile.com/b/33ouGt/IHz0)

105. [Deans, Z.C.; Butler, R.; Cheetham, M.; Dequeker, E.M.C.; Fairley, J.A.; Fenizia, F.; Hall, J.A.; Keppens, C.; Normanno, N.; Schuuring, E.; et al. IQN path ASBL report from the first European cfDNA consensus meeting: expert opinion on the minimal requirements for clinical ctDNA testing. *Virchows Arch.* **2019**, *474*, 681–689.](http://paperpile.com/b/33ouGt/MDCQ)

106. [miRNeasy Serum/Plasma Kit Available online:](http://paperpile.com/b/33ouGt/rUMK) <https://www.qiagen.com/us/products/diagnostics-and-clinical-research/sample-processing/mirneasy-serumplasma-kit/#orderinginformation> [(accessed on Sep 6, 2020).](http://paperpile.com/b/33ouGt/rUMK)

107. [miRNeasy 96 Advanced QIAcube HT Kit Available online:](http://paperpile.com/b/33ouGt/pUYH) <https://www.qiagen.com/dk/products/discovery-translational-research/dna-rna-purification/rna-purification/mirna/mirneasy-96-advanced-qiacube-ht-kit/?clear=true#orderinginformation> [(accessed on Sep 6, 2020).](http://paperpile.com/b/33ouGt/pUYH)

108. [Plasma/Serum Circulating and Exosomal RNA Purification Kit (Slurry Format) (Cat. 42800) Available online:](http://paperpile.com/b/33ouGt/xkxT) <https://norgenbiotek.com/product/plasmaserum-circulating-and-exosomal-rna-purification-kit-slurry-format> [(accessed on Sep 6, 2020).](http://paperpile.com/b/33ouGt/xkxT)

109. [Takano, S.; Hu, Q.; Amamoto, T.; Refinetti, P.; Mimori, K.; Funatsu, T.; Kato, M. Extraction of cell-free DNA from urine, using polylysine-coated silica particles. *Anal. Bioanal. Chem.* **2017**, *409*, 4021–4025.](http://paperpile.com/b/33ouGt/V4L2)

110. [Kato, M.; Fujita, Y.; Iizuka, T.; Nozaki, K.; Takano, S.; Funatsu, T.; Sano, Y.; Murayama, S.; Karasawa, K. Extraction of urinary cell-free DNA by using triamine-modified silica particles for liquid biopsy. *Anal. Bioanal. Chem.* **2020**, *412*, 5647–5652.](http://paperpile.com/b/33ouGt/RqRD)

111. [Oreskovic, A.; Brault, N.D.; Panpradist, N.; Lai, J.J.; Lutz, B.R. Analytical Comparison of Methods for Extraction of Short Cell-Free DNA from Urine. *J. Mol. Diagn.* **2019**, *21*, 1067–1078.](http://paperpile.com/b/33ouGt/gXbO)

112. [Lee, E.Y.; Lee, E.-J.; Yoon, H.; Lee, D.H.; Kim, K.H. Comparison of Four Commercial Kits for Isolation of Urinary Cell-Free DNA and Sample Storage Conditions. *Diagnostics (Basel)* **2020**, *10*, doi:](http://paperpile.com/b/33ouGt/p8Hx)[10.3390/diagnostics10040234](http://dx.doi.org/10.3390/diagnostics10040234)[.](http://paperpile.com/b/33ouGt/p8Hx)

113. [Streleckiene, G.; Reid, H.M.; Arnold, N.; Bauerschlag, D.; Forster, M. Quantifying cell free DNA in urine: comparison between commercial kits, impact of gender and inter-individual variation. *Biotechniques* **2018**, *64*, 225–230.](http://paperpile.com/b/33ouGt/rAZo)

114. [Augustus, E.; Van Casteren, K.; Sorber, L.; van Dam, P.; Roeyen, G.; Peeters, M.; Vorsters, A.; Wouters, A.; Raskin, J.; Rolfo, C.; et al. The art of obtaining a high yield of cell-free DNA from urine. *PLoS One* **2020**, *15*, e0231058.](http://paperpile.com/b/33ouGt/LIO3)

115. [Martínez-Fernández, M.; Paramio, J.M.; Dueñas, M. RNA Detection in Urine: From RNA Extraction to Good Normalizer Molecules. *J. Mol. Diagn.* **2016**, *18*, 15–22.](http://paperpile.com/b/33ouGt/a758)

116. [QIAsymphony DSP Circulating DNA Kit Available online:](http://paperpile.com/b/33ouGt/qVwe) <https://www.qiagen.com/au/products/discovery-and-translational-research/dna-rna-purification/dna-purification/cell-free-dna/qiasymphony-dsp-circulating-dna-kit/#orderinginformation> [(accessed on Sep 6, 2020).](http://paperpile.com/b/33ouGt/qVwe)

117. [Zhao, A.; Péoc’h, M.; Cottier, M.; Genin, C.; Mottet, N.; Li, G. Cell-free RNA content in urine as a possible molecular diagnostic tool for clear cell renal cell carcinoma. *Int. J. Cancer* **2015**, *136*, 2610–2615.](http://paperpile.com/b/33ouGt/LkRP)

118. [[No title] Available online:](http://paperpile.com/b/33ouGt/ZeW7) <https://files.zymoresearch.com/protocols/_d4076_quick-cfdna_serum_plasma_kit.pdf> [(accessed on Sep 6, 2020).](http://paperpile.com/b/33ouGt/ZeW7)

119. [Reedy, C.R.; Price, C.W.; Sniegowski, J.; Ferrance, J.P.; Begley, M.; Landers, J.P. Solid phase extraction of DNA from biological samples in a post-based, high surface area poly(methyl methacrylate) (PMMA) microdevice. *Lab Chip* **2011**, *11*, 1603–1611.](http://paperpile.com/b/33ouGt/wf7D)

120. [Pekin, D.; Skhiri, Y.; Baret, J.-C.; Le Corre, D.; Mazutis, L.; Salem, C.B.; Millot, F.; El Harrak, A.; Hutchison, J.B.; Larson, J.W.; et al. Quantitative and sensitive detection of rare mutations using droplet-based microfluidics. *Lab Chip* **2011**, *11*, 2156–2166.](http://paperpile.com/b/33ouGt/58mR)

121. [Kim, S.; De Jonghe, J.; Kulesa, A.B.; Feldman, D.; Vatanen, T.; Bhattacharyya, R.P.; Berdy, B.; Gomez, J.; Nolan, J.; Epstein, S.; et al. High-throughput automated microfluidic sample preparation for accurate microbial genomics. *Nat. Commun.* **2017**, *8*, 13919.](http://paperpile.com/b/33ouGt/diea)

122. [Bienvenue, J.M.; Legendre, L.A.; Ferrance, J.P.; Landers, J.P. An integrated microfluidic device for DNA purification and PCR amplification of STR fragments. *Forensic Sci. Int. Genet.* **2010**, *4*, 178–186.](http://paperpile.com/b/33ouGt/UKYL)

123. [Oblath, E.A.; Henley, W.H.; Alarie, J.P.; Ramsey, J.M. A microfluidic chip integrating DNA extraction and real-time PCR for the detection of bacteria in saliva. *Lab Chip* **2013**, *13*, 1325–1332.](http://paperpile.com/b/33ouGt/UTRg)

124. [Gai, C.; Camussi, F.; Broccoletti, R.; Gambino, A.; Cabras, M.; Molinaro, L.; Carossa, S.; Camussi, G.; Arduino, P.G. Salivary extracellular vesicle-associated miRNAs as potential biomarkers in oral squamous cell carcinoma. *BMC Cancer* **2018**, *18*, 1–11.](http://paperpile.com/b/33ouGt/LOqt)

125. [Bronkhorst, A.J.; Ungerer, V.; Holdenrieder, S. Comparison of methods for the quantification of cell-free DNA isolated from cell culture supernatant. *Tumour Biol.* **2019**, *41*, 1010428319866369.](http://paperpile.com/b/33ouGt/TZep)

126. [Tina Draškovič Isolation of Cell-Free DNA from Seminal Fluid. *JPP* **2017**, *5*, doi:](http://paperpile.com/b/33ouGt/iGgR)[10.17265/2328-2150/2017.08.010](http://dx.doi.org/10.17265/2328-2150/2017.08.010)[.](http://paperpile.com/b/33ouGt/iGgR)

127. [Lee, J.S.; Hur, J.Y.; Kim, I.A.; Kim, H.J.; Choi, C.M.; Lee, J.C.; Kim, W.S.; Lee, K.Y. Liquid biopsy using the supernatant of a pleural effusion for EGFR genotyping in pulmonary adenocarcinoma patients: a comparison between cell-free DNA and extracellular vesicle-derived DNA. *BMC Cancer* **2018**, *18*, 1236.](http://paperpile.com/b/33ouGt/WaGH)

128. [Hummelink, K.; Muller, M.; Linders, T.C.; van der Noort, V.; Nederlof, P.M.; Baas, P.; Burgers, S.; Smit, E.F.; Meijer, G.A.; van den Heuvel, M.M.; et al. Cell-free DNA in the supernatant of pleural effusion can be used to detect driver and resistance mutations, and can guide tyrosine kinase inhibitor treatment decisions. *ERJ Open Res* **2019**, *5*, doi:](http://paperpile.com/b/33ouGt/VA5t)[10.1183/23120541.00016-2019](http://dx.doi.org/10.1183/23120541.00016-2019)[.](http://paperpile.com/b/33ouGt/VA5t)

129. [Hui, L.; Tong, S.; Kaitu’u-Lino, T.J.; Hannan, N.J. A comparison of sample collection methods for quantifying cell-free fetal neurodevelopment transcripts in amniotic fluid. *BMC Res. Notes* **2016**, *9*, 335.](http://paperpile.com/b/33ouGt/AdeJ)
